# Editorial: The role of bacteriophages in *Salmonella* diversity, pathogenicity and control

**DOI:** 10.3389/fmicb.2025.1591189

**Published:** 2025-03-27

**Authors:** Anisha M. Thanki, Steven P. T. Hooton, Parameth Thiennimitr, Janet Y. Nale

**Affiliations:** ^1^Becky Mayer Centre for Phage Research, University of Leicester, Leicester, United Kingdom; ^2^Department of Microbiology, Faculty of Medicine, Chiang Mai University, Chiang Mai, Thailand; ^3^School of Veterinary Medicine, Centre for Epidemiology and Planetary Health, Scotland's Rural College, Inverness, United Kingdom

**Keywords:** *Salmonella*, Phage (bacteriophage), phage therapy, *Salmonella* serovar, *Salmonella* infection models

## Introduction

*Salmonella* causes gastroenteritis in humans and is spread through contaminated water and food, particularly undercooked poultry and pork (Shaji et al., 2023; Soliani et al., 2023; Popa and Papa, 2021). Control measures include preventive measures such as vaccines and hygiene practices, pathogen surveillance, animal pre-slaughter interventions, and antibiotic use (Majowicz et al., 2010; Nale et al., 2023; Arguello et al., 2013; Viltrop et al., 2023). However, *Salmonella* infection remains high and several serovars exhibit multidrug resistance, such as to colistin (Zhang et al., 2022). Effective control requires innovative and more robust strategies targeting key serovars, and understanding the virulence determinants influencing their pathogenicity and evolution (Nale et al., 2023; Andrews et al., 2024).

Bacteriophages (phages) are parasitic viruses that specifically infect bacteria and can either lyse their hosts or contribute to gene transfer, thereby impacting strain emergence (Nale et al., 2023; Andrews et al., 2024). These interactions can be harnessed to control *Salmonella* proliferation or to study their role in disease outbreaks. To disseminate research in this area, this Research Topic focused on the isolation and characterization of *Salmonella* phages, their genetic diversity, host interactions, therapeutic potential, synergy with antibiotics, formulation for optimal stability, delivery and activity, and role in horizontal gene transfer. The six original articles published in this Research Topic are outlined below and summarized in Table 1.

**Table 1:** Summary of studies published in this Research Topic and their outcomes.

**Studies**	**Target serovars**	**Phages tested**	**Infection systems/models**	**Therapeutic effectiveness/outcome**
Juliet et al.	Not specified	Phage vB_SalP_792	Chicken egg white and yolk, and biofilm	The phage was more stable and significantly reduced bacteria in egg white than in egg yolk. There was also a notable decrease in biofilms after treatment with the phage *in vitro*
Sevilla-Navarro et al.	*S*. Infantis	vB_Si_CECAV_FGS009; vB_Si_CECAV_FGS017; vB_Si_CECAV_FGS029 and vB_Si_CECAV _FGS030	Broiler farms associated with cleaning and disinfection protocols	Phage treatment reduced *S*. Infantis load on the farms and with cleaning protocols, the bacterium was undetected
Deng et al.	*S*. Pullorum	YSP2	Identified tail fiber protein, 35Q of YSP2 by mass spectrometry, protein and comparative genomic analyses, and expression assays	Tail fiber protein, 35Q of YSP2 interacted with the outer membrane protein receptor on *S*. Pullorum surfaces
Battistelli et al.	*S*. Infantis	SaI_NFG_5581 and SaI_NFG_5577	Stability, growth kinetics and infectivity profiles of the phages *in vitro*.	Phage (single, sequential and cocktail)-treated cultures showed reduced bacterial counts compared to the control within the 24-h treatment period
Luo et al.	*S*. Sendai, *S*. Enteritidis, *S*. Equine, *S*. Tennessee, *S*. Paratyphi, *S*. Africana, *S*. Typhimurium, *S*. Paratyphi A and *S*. Paratyphi B	Phage vB-SeS-01	*Ex vivo* using HeLa cells	The treatment of *S*. Sendai- and *S*. Enteritidis-infected HeLa cells with encapsulated liposomal and free phages showed a reduction in intracellular microbial load compared to control.
Torkashvand et al.	*S*. Enteritidis	vB_SenS_TUMS_E4 (E4), vB_SenS_TUMS_E15 (E15), and vB_SenS_TUMS_E19 (E19)	Human foreskin fibroblast cell line, biofilm and food- chicken breast, cherry tomato, quail eggshell, and lettuce	Undetected toxicity on the human cell line. Therapeutic efficacy of the individual phages and in combination was observed in biofilms and on the food sources

In the study by Juliet et al., the authors reported the isolation from sewage and genomic characterization of a *Salmonella* phage, vB-SalP_792. The essential properties (host range, morphology, pH, and thermal stability) of the phage for use as a food biocontrol agent were tested. Therapeutic trials showed better reduction in bacterial load in egg white compared to egg yolk, and on established biofilms post-phage treatment. This study supports the potential application of the lytic phage vB-SalP_792 against multidrug-resistant *Salmonella*, especially to address food safety concerns.

The next published study by Sevilla-Navarro et al., reported on the effectiveness of four novel broad host range lytic phages for *S*. infantis control in 10 commercial broiler farms. The phages were tested in combination with cleaning and disinfection (with a combination of glutaraldehyde, quaternary ammonium compounds, and peroxides) protocols. Prior to treatment, *Salmonella* was recovered from all hen houses. However, a significant reduction in bacterial recovery was observed after the first and second phage applications, and between cleaning procedure steps which greatly improved disinfection. *S*. infantis was not detected in hen houses post-treatment, which improved conventional cleaning and disinfection protocols. The data support the application of the phages to control *S*. Infantis in broiler/poultry farms.

In the work by Deng et al., the authors determined the receptor binding protein of phage YSP2 on its target *S*. Pullorum host strain. The tail fiber protein, 35Q of YSP2 was identified by mass spectrometry and comparative genomic analysis to be the binding receptor. When expressed using an *Escherichia coli* expression system and the efficiency analyzed using ELISA assays and adsorption experiments, the 35Q protein was shown to interact with the outer membrane protein receptor on the host surface. By identifying the phage receptor, these data provide useful knowledge into how *Salmonella* phages interact with their bacterial hosts and their potential application in diagnostics and therapeutics.

Two virulent phages were isolated from broiler feces and characterized by Battistelli et al. The phages were genetically different, lacked lysogeny and virulence genes, and were found to be stable at temperatures up to 50°C and pH 6–10. A combination of the phages, one phage added before the other or simultaneously, significantly reduced the bacterial load within 2 h *in vitro*. However, bacterial regrowth was observed from this time until the end of the assay (24 h), although the treatment showed a better bacterial reduction compared to the control. The observed bacterial regrowth could be associated with the development of phage resistance and a probable lack of complementation by the two phages to control the phage-resistant bacteria as shown in previous studies (Nale et al., 2023, 2021). Although the phages showed some therapeutic potential *in vitro*, it is clear that the phage cocktail can be improved by including other active phages in the mix to mitigate phage resistance. This strategy can be used as a pre-slaughter intervention step to curtail the growth of *S*. infantis in chickens.

To ensure optimal delivery to specific target sites, especially within epithelial cells, where *Salmonella* can invade to cause systemic infection, the report by Luo et al. focused on liposomal encapsulation of a novel phage. The *S. enterica* phage, vB-SeS-01 belonging to *Guernseyvirinae* was isolated from soils collected from a chicken farm. The phage showed low host specificity, lysing only nine of the 24 isolates tested but exhibited a burst of 115 PFU/mL and high pH (4–13) and thermal (4–80°C) stability. A liposomal encapsulated phage with average particle size (177.03–205.13 nm) and PDI (0.096–0.199) was optimized, and sonication was found to negatively impact encapsulation efficiency. The stability of the phage in intestinal simulating fluid for up to 120 min was better for encapsulated complexes compared to the free phages. Dosages of encapsulated phages were determined through toxicity assays using HeLa cells and intracellular antibacterial effects were found to be superior with encapsulated phages compared to free ones. Although transcriptional analyses revealed that both encapsulated and free phages reduced the infection level to one-third focusing on the response of TNF-α, only the liposome complexes reduced the transcription level of IL-8 in HeLa cell lines infected with *S*. Sendai and *S*. Enteritidis. The data support the use of encapsulation to further improve phage stability and activity.

In the study by Torkashvand et al., a novel phage cocktail consisting of three previously characterized lytic phages was investigated to determine its therapeutic efficacy and safety against *S*. enteritidis. High titer phages were produced and through a colourimetric MTT [3-(4,5-dimethylthiazol-2-yl)-2,5 diphenyl tetrazolium bromide] assay with a human foreskin fibroblast cell line, high cell viability was observed within 24 h. This indicates the safety of the phages. A time-to-kill assay was conducted *in vitro* with *Salmonella* planktonic cells treated with phages at different multiplicities of infection (number of phages exposed to bacteria). The data showed ~4 log reduction at 10 h post-exposure; however, regrowth was observed 4 h later indicating potential phage resistance. Exposing *Salmonella* biofilms to each of the three phages showed significant biofilm reduction, and a similar effect was observed when the phages were combined with ampicillin. Furthermore, the phage cocktail inhibited *Salmonella* growth on chicken breast meat, cherry tomatoes, quail eggshells, and lettuce within 15 min exposure at 4°C and 25°C. The examined phages clearly have therapeutic potential to reduce *Salmonella* contamination in food.

## Conclusion

*Salmonella*, an important zoonotic pathogen, causes gastrointestinal infections in humans. Several serovars are resistant to various frontline antibiotics highlighting the need for urgent and more effective alternative control strategies. The recently completed Research Topic published original research articles in the areas of phage therapeutic application, targeting phage isolation and phenotypic and genomic characterization, testing in various infection models and formulation to enhance therapeutic activity against different serovars.
